# Integrating Real-Time Air Quality Monitoring, Ecological Momentary Assessment, and Spirometry to Evaluate Asthma Symptoms: Usability Study

**DOI:** 10.2196/60147

**Published:** 2024-10-10

**Authors:** Barbara Polivka, Kathryn Krueger, Olivia Bimbi, Luz Huntington-Moskos, Sharmilee Nyenhuis, Emily Cramer, Kamal Eldeirawi

**Affiliations:** 1 School of Nursing University of Kansas Kansas City, KS United States; 2 College of Nursing University of Illinois at Chicago Chicago, IL United States; 3 School of Nursing University of Louisville Louisvlle, KY United States; 4 University of Chicago Medicine Chicago, IL United States; 5 School of Medicine University of Missouri-Kansas City Kansas City, MO United States

**Keywords:** indoor air quality, asthma, real-time assessment, EMA, ecological momentary assessment, mobile phone, monitoring, air quality, real time, spirometry, acceptability, usability, residential toxins, volatile organic compounds, VOC, adult, female, women, college student

## Abstract

**Background:**

Individuals are exposed to a variety of indoor residential toxins including volatile organic compounds and particulates. In adults with asthma, such exposures are associated with asthma symptoms, asthma exacerbations, and decreased lung function. However, data on these exposures and asthma-related outcomes are generally collected at different times and not in real time. The integration of multiple platforms to collect real-time data on environmental exposure, asthma symptoms, and lung function has rarely been explored.

**Objective:**

This paper describes how adults with asthma perceive the acceptability and usability of three integrated devices: (1) residential indoor air quality monitor, (2) ecological momentary assessment (EMA) surveys delivered via a smartphone app, and (3) home spirometry, over 14 days.

**Methods:**

Participants (N=40) with uncontrolled asthma were mailed the Awair Omni indoor air quality monitor, ZEPHYRx home spirometer, and detailed instructions required for the in-home monitoring. The air quality monitor, spirometer, and EMA app were set up and tested during a videoconference or phone orientation with a research team member. Midway through the 14-day data collection period, participants completed an interview about the acceptability of the study devices or apps, instructional materials provided, and the setup process. At the end of the 14-day data collection period, participants completed a modified System Usability Scale. A random sample of 20 participants also completed a phone interview regarding the acceptability of the study and the impact of the study on their asthma.

**Results:**

Participants ranged in age from 26 to 77 (mean 45, SD 13.5) years and were primarily female (n=36, 90%), White (n=26, 67%), college graduates (n=25, 66%), and residing in a single-family home (n=30, 75%). Most indicated that the air quality monitor (n=23, 58%), the EMA (n=20, 50%), and the spirometer (n=17, 43%) were easy to set up and use. Challenges with the EMA included repetitive surveys, surveys arriving during the night, and technical issues. While the home spirometer was identified as a plausible means to evaluate lung function in real time, the interpretation of the readings was unclear, and several participants reported side effects from home spirometer use. Overall, the acceptability of the study and the System Usability Scale scores were high.

**Conclusions:**

The study devices were highly acceptable and usable. Participant feedback was instrumental in identifying technical challenges that should be addressed in future studies.

## Introduction

Individuals are exposed to indoor residential toxins including volatile organic compounds (VOCs) and particulates [1]. Common indoor sources of VOCs include cleaning or disinfecting products, personal care products, air fresheners, fragrances, and pesticides [1-4]. Residential sources of particulates include vacuum cleaning, cooking, tobacco smoke, wood-burning fireplaces, dust, candles, and animal dander [4-7]. While indoor residential exposure to VOCs and particulates has been associated with asthma symptoms, data on asthma exacerbations, lung function, indoor VOCs and fine particulates, and pulmonary or asthma symptoms are generally collected at different times, or researchers rely solely on electronic health records or survey data [5,8-11]. The collection of real-time data on environmental exposures, asthma symptoms, and lung function has rarely been explored. In their 2020 study, Turner et al [12] had adolescents wear personal ultrafine particulate monitors for 3 hours per day for 7 days. In addition, participants completed home spirometry 5 times per day and ecological momentary assessment (EMA) surveys on a smartphone to determine respiratory symptoms throughout the day. While it was possible for Turner et al [12] to retrospectively assess associations between lung function and exposures within 30 minutes, real-time determination of exposure and its pulmonary impact was not possible.

We designed a feasibility study to capture residential indoor air quality in real time and thus examine the real-time impact of residential indoor air quality on asthma symptoms and lung function in a sample of adults with uncontrolled asthma [13]. A primary purpose of the feasibility study was to determine the acceptability and usability of (1) real-time residential indoor air quality monitoring along with EMA to evaluate asthma symptoms in response to poor residential air quality and (2) using home spirometry to monitor lung function in this population.

Although the technology for low-cost, residential, consumer-grade, continuous indoor air quality monitors has evolved [14-18] and user-friendly sensors are often advocated for indoor residential use [15], few studies to date have evaluated the acceptability and usability of these sensors from the user’s perspective to further understand the implementation potential. Moore et al [19] used 3 Dylos air quality monitors to assess levels of particulates 2.5 microns or less in diameter (PM_2.5_) in 6 homes with SMS text messages delivered when a spike in the PM_2.5_ levels occurred. A tablet visualization interface allowed for viewing data trends across time. Participants indicated they appreciated knowing about the indoor air quality in various rooms, how their air quality changed over time, and how activities such as cooking impacted their home indoor environment. However, engagement over time (up to 40 weeks) decreased.

Home-based spirometry provides a means for obtaining timely information regarding lung function remotely, thus facilitating self-management and minimizing the need for clinic or hospital visits [20]. Acceptability and usefulness of home spirometry for individuals with chronic obstructive pulmonary disease, interstitial lung disease, cystic fibrosis, and asthma were found to be high, with most indicating the device was easy to use, easy to set up, cost-effective, empowering, and convenient [21-23]. While motivation to exercise to improve lung function readings was also noted as a benefit by adults with cystic fibrosis, a concern raised during interviews was distrust of technology [22].

EMA has been used in a variety of studies including those addressing mental health, substance abuse, diet, weight loss, smoking, well-being, pain, eating disorders, physical activity, sleep, fatigue, and asthma [24-28]. Participants in these studies reported strengths of EMA surveys including ease of use, simplicity, fun to use, a graphic user interface that includes easy-to-follow prompts, survey items that increase awareness of personal behaviors, and reminders to respond to prompts and complete certain behaviors such as taking medication [29]. Identified weaknesses of EMA include too many surveys, tediousness, technical issues, repetitiveness, lack of customization, unclear survey items, questionable timing of surveys, easily missed surveys, lack of technological features such as text-to-speech capability, and the length of some surveys [24,30-34].

Acceptability and usability of home spirometry, EMA, and residential indoor air quality monitors are generally high. However, evidence is lacking on the acceptability and usability of these devices or apps when used simultaneously by adults with asthma. The purpose of this paper is to describe how adults with asthma perceive the acceptability and usability of three integrated devices: (1) residential indoor air quality monitor, (2) EMA delivered via smartphones, and (3) home spirometry, over 14 days.

## Methods

### Study Design

Participants were recruited from a pool of adults with asthma who previously completed a survey concerning the COVID-19 pandemic [35], who indicated they were interested in future research opportunities, and who reported that they used disinfectants or cleaning products at least 5 times per week. Inclusion criteria for this study were adults 18 years old or older who own a smartphone, have a Wi-Fi or wireless internet connection in their home, and have uncontrolled asthma (Asthma Control Test <20 or asthma exacerbation in the past year). The protocol for this study has been previously published [13]. A total of 40 participants completed the 2-week, in-home study between April 2022 and March 2023. Participants were mailed the indoor air quality monitor, home spirometer, and detailed instructions for in-home testing. Prior to the start of the study, instructions to deploy each device (air quality monitor, spirometer, and EMA app) were developed with input from a community advisory board (CAB). The CAB recommended that the instructions for each device include detailed step-by-step directions and clear pictures of the setup. For example, based on recommendations from the CAB, we added screenshots of the spirometer app setup screen with added text explaining what and where information was needed. The CAB was compensated for providing feedback to the research team.

**Figure 1:**
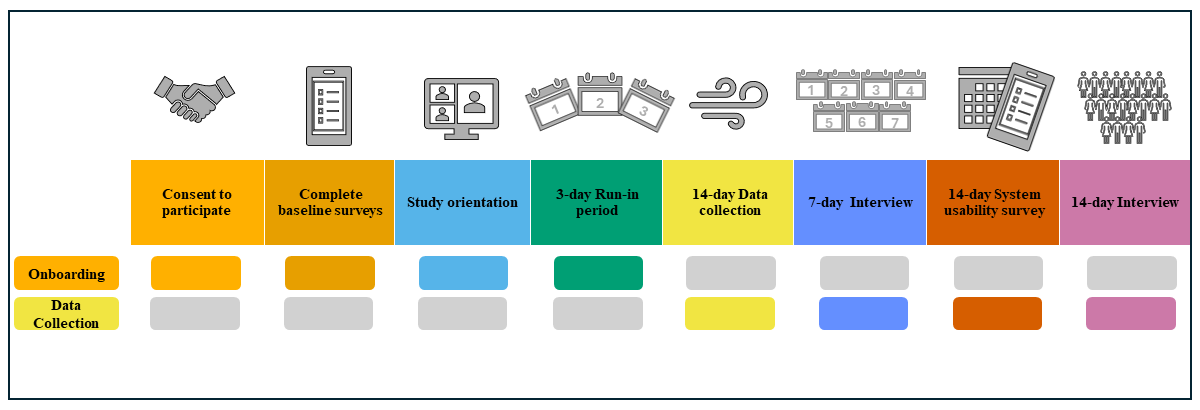
Study onboarding and data collection timeline.

A live, one-on-one orientation session with a research team member via videoconference or phone was completed, during which instructions for the study were reviewed and the air quality monitor, spirometer and app, and the EMA app were set up and tested (Figure 1). Orientation sessions averaged 1 hour. Participants then completed a 3-day, run-in period to become familiar with the apps and equipment and to ensure data from each device were transmitted to its corresponding dashboard. Following the 3-day, run-in period, participants completed the 14-day data collection period. The research team monitored all data transmitted to the dashboards throughout the run-in and data collection periods and conducted troubleshooting as necessary when problems were identified (ie, Wi-Fi connectivity issues, needing to log back into the EMA or spirometry app).

Midway through the 14-day data collection period, participants completed a 7-day interview and were asked about the acceptability of the air quality monitor, spirometer, and EMA. Participants were also queried regarding the instructional materials provided, the orientation to the study, and the setup process. Usability of the study devices was assessed using a modified System Usability Scale (SUS) [36]. All participants were emailed a link to the modified SUS on REDCap (Research Electronic Data Capture; Vanderbilt University) at the end of the 14-day data collection period. In addition, a random sample of 20 participants was selected to complete a phone interview. In the phone interview, participants were again queried about the acceptability of the study devices, the overall study, the impact of the study on their asthma, and suggestions for improving the study. The 7 and 14-day interviews were recorded.

### Ethical Considerations

The study was approved by the institutional review boards at the University of Kansas Medical Center (00145830), the University of Louisville (21.0466), the University of Illinois Chicago (2020-0851), and the University of Chicago (22-0767) prior to the conduct of the study. Participants provided e-consent for the study prior to completing the REDCap baseline survey. Data were deidentified prior to analysis. Participants kept the spirometer as an incentive (US $125 value).

### Study Devices

**Figure 2:**
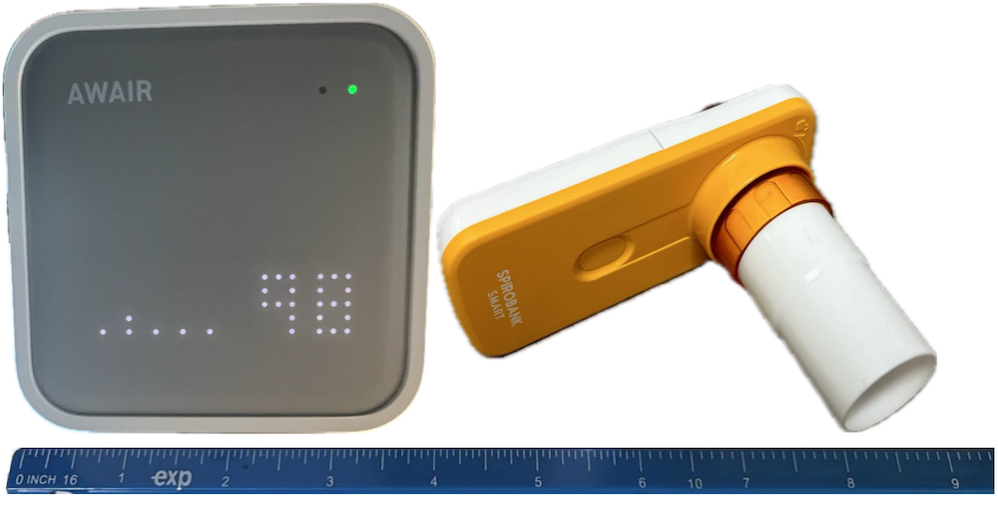
Images of the (A) Awair Omni indoor air quality monitor and (B) the ZephyRx home spirometer. A ruler is included to illustrate the approximate sizes of the devices.

The commercially available Awair Omni indoor air quality monitor measured total VOCs (TVOCs) and PM_2.5_ in participants’ home environments. The air quality monitor plugs into a standard electrical outlet, has an 8-hour internal battery backup, and is Wi-Fi enabled (Figure 2). Participants were instructed to place the air quality monitor in the room where they spend most of their time at home. Specifically, participants were asked to place the air quality monitor in a central location, 3 to 6 feet off the ground, and as far as possible from a window or door. Participants were also asked to move the monitor into the room where they slept at night.

The ZEPHYRx Spirometer system includes a handheld Bluetooth spirometer (MIR Spirobank Smart) and a mobile app (Figure 2). The research team developed the instruction guide and provided step-by-step information on how to use and care for the spirometer. Participants were encouraged to use the nose clip included with their spirometer when they were completing pulmonary function testing. A trained research team member initially coached each participant through the spirometry procedures. Participants were asked to use their spirometer each morning and when prompted by an EMA survey triggered by elevated TVOC or PM_2.5_ levels detected by the air quality monitor.

**Figure 3:**
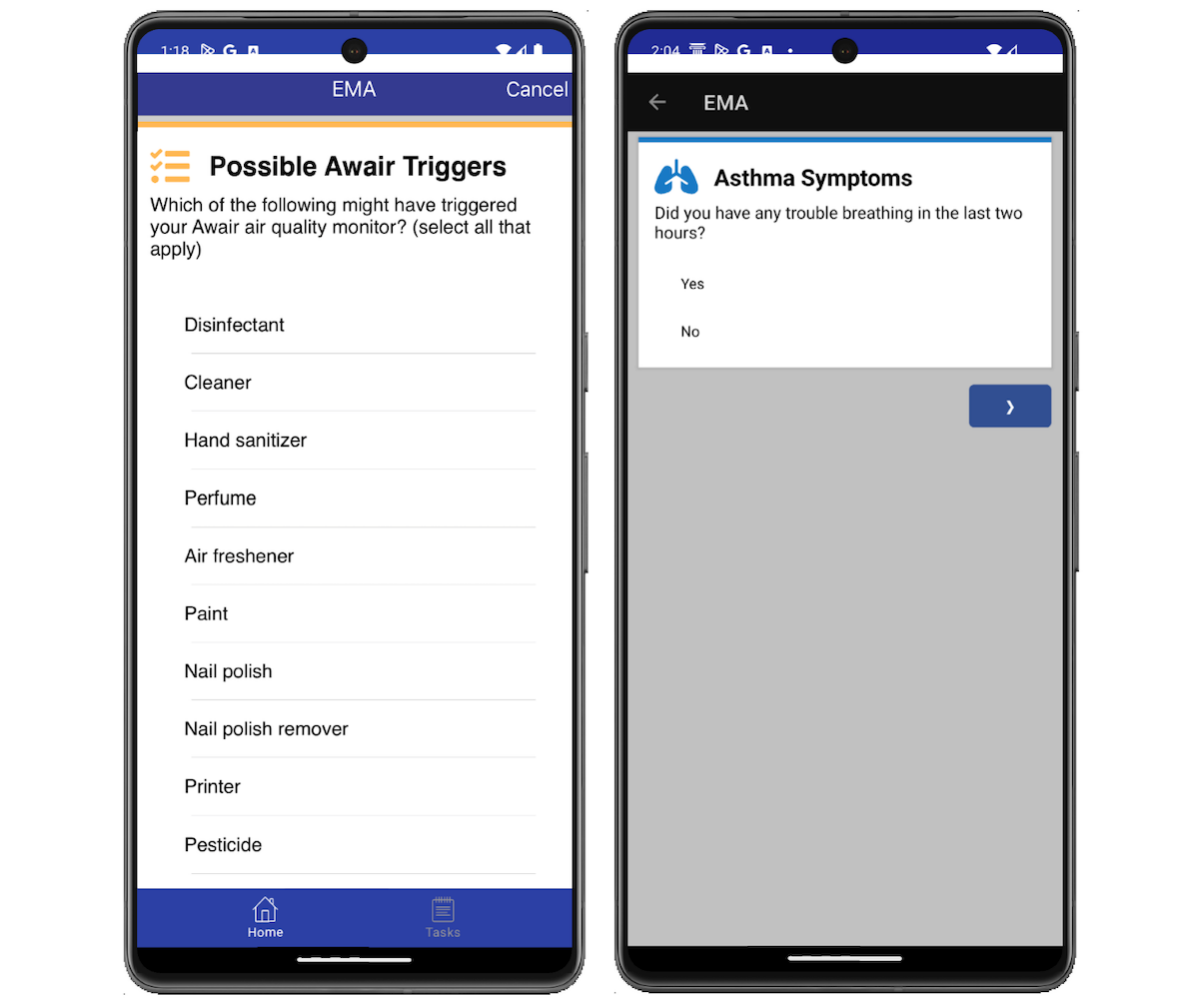
Screenshots of EMA survey items querying (A) possible sources of the triggered the air quality event and (B) asthma symptoms. EMA: ecological momentary assessment.

The PiLR EMA (MEI Research) app was used to gather real-time data regarding residential air quality events and real-time asthma symptoms. Participants received EMA surveys on their smartphones each morning, randomly twice each day, and when elevated TVOC or PM_2.5_ levels were detected by the air quality monitor (Figure 3). Specifically, each morning participants received an EMA survey concerning nighttime asthma symptoms; the survey included a reminder to use their spirometer. EMA surveys addressing the use of cleaners, disinfectants, or hand sanitizers in the home during the previous 2 hours along with current asthma symptoms occurred randomly twice each day between 6 AM and 10 PM. Finally, to detect real-time TVOC or PM_2.5_ residential exposure and concomitant asthma symptoms, the publicly available application programming interface of the Awair Omni air quality monitor was used to develop the EMA software that enabled an EMA survey to be sent to the participant immediately at specific target points. An EMA survey was sent when levels of indoor residential TVOCs or PM_2.5_ were detected above a specific threshold level—TVOCs ≥333 ppb and PM_2.5_ ≥15 µg/m^3^. The EMA survey noted that the participant’s air quality monitor detected an air quality change in their residence. Participants were initially asked whether they were home. For those indicating they were at home, a drop-down list of possible sources for the air quality change was presented (eg, disinfectant use, cooking, pets, and burning candles). Participants were asked to identify the potential sources of the air quality change from a list of 18 options (eg, disinfectant, perfume, and cooking) that included “I don’t know” and an open-ended other option. In addition, participants could take a picture of the potential source of the air quality change and upload it to the EMA survey. The EMA survey also asked participants to indicate if they were currently having any asthma symptoms. Participants were prompted to use their spirometer at that time as well.

### Instruments

Demographic data such as age, sex, race, education, employment status, and residence were collected at baseline using REDCap. The modified SUS [36-38] included 3 subscales, the air quality monitor SUS, the spirometer SUS, and the EMA SUS, to specifically capture the usability of each device or app (eg, I thought the Awair air quality monitor was easy to use). Modifications to the original 10-item SUS included specifying the time frame as “over the last two weeks” and querying if each device was well integrated into the study. The air quality and the EMA SUS subscales each had 11 items with the 1 additional item asking if the participant would recommend that device to others with asthma. The SUS EMA included 13 items; participants were also asked if they found the EMA surveys unnecessarily repetitive, time-consuming, and arriving at an inconvenient time. One additional item was added at the end of the survey that addressed the overall integration of the various study components. Response options ranged from 1= strongly agree to 5= strongly disagree for all survey items. An open-ended item at the end of each subscale allowed participants to write comments regarding what they liked or disliked about each study device.

### Data Management and Analysis

Quantitative data were downloaded from REDCap into SPSS Statistics (IBM) [39]. Demographic data were analyzed using descriptive statistics. A composite SUS score for each subscale was calculated using the method described by Brooke [36]. To create SUS subscale scores, individual items were recoded to a 0-4 scale with higher values being more positive and summed to create scale scores. Negatively worded items were reverse-coded. Raw summed scores for the air quality monitor and spirometer subscales (11 items each) were multiplied by a multiplier of 2.27 to convert to a 0 to 100 scale; the EMA subscale (13 items) raw summed score was multiplied by a multiplier of 1.92 to convert to a 0 to 100 scale; and the overall SUS item score addressing integration of study components was multiplied by a factor of 25.

Open-ended responses to SUS items were downloaded into Dedoose software (SocioCultural Research Consultants). Participants’ 7 and 14-day interviews were transcribed, validated for accuracy, and added to Dedoose. Themes related to the acceptability and usability of each of the devices were determined based on written responses to open-ended SUS items and the transcripts from the 7 and 14-day interviews and validated independently by 2 research team members.

## Results

### Overview

Participants (N=40) were primarily female (n=36, 90%), college graduates (n=25, 66%), employed (n=30, 75%), owners of their home (n=27, 67.5%), and residing in a single-family home (n=30, 75%), and they were from 18 (36%) different US states. Most participants indicated they were non-Hispanic (n=38, 95%) and White (n=26, 67%). A total of 5 (13%) participants indicated Black or African American race, 4 (10%) reported their race as American Indian or Alaskan Native, and 2 (5%) indicated Asian race. Participants ranged in age from 26 to 77 (mean 45, SD 13.5) years.

### Indoor Air Quality Monitor

Study participants reported that the indoor air quality monitor was easy to use (n=23, 58%) and educational (n=6, 15%), with 1 (3%) participant commenting that they “started noticing trends that set off the air quality change surveys” (Table 1). The real-time reporting of air quality results was also mentioned by several participants (n=6, 15%) as a benefit of the monitor. A total of 4 (10%) participants described confusion at times when the air quality monitor indicated an elevated measurement, but they could not identify the source of the air quality change or what the alerts meant. Three (8%) participants reported that the device “was very sensitive” or “goes off a lot.” Two (5%) participants noted that the brightness from the light on the front display of the air quality monitor was an issue at night while trying to sleep. In addition, several technical issues were reported by 7 (18%) participants, including trouble connecting the device to their home Wi-Fi system, difficulties connecting the power cord, and needing to have the device continuously plugged into an outlet.

**Table 1:** Themes and quotes related to participant experiences with the indoor air quality monitor.

Indoor air quality monitor themes	Indoor air quality monitor quotes
Easy to use	“This tool was very easy to use.” [ID 5] “It’s almost unnoticeable now...cause it’s just sitting there...it’s not needing any maintenance or anything.” [ID 89]
Real-time results	“It has been really interesting to actually watch it.” [ID 27] “I liked being able to monitor my air quality.” [ID120] “It’s nice to know when my air quality changed.” [ID 37]
Educational	“I started noticing trends that set off the air quality change surveys, so I altered my behavior slightly.” [ID 58] “It heightened my awareness.” [ID 73]
Notification timing	“It goes off a lot.” [ID 17] “It was very sensitive.” [ID 101]
Clarity of results	“I don’t always know what has triggered it.” [ID 17] “It’ll sometimes register an event when nothing is really happening.” [ID35]
Technical issues	“I don’t know what the number means.” [ID 71] “I had a very difficult time getting the Awair to stay connected to my Wi-Fi.” [ID 11] “Would like a longer battery life so I wouldn’t have to have it plugged in all the time.” [ID 91] “I had a bit of trouble with the power cord.” [ID 11]
Bright display at night	“The display was bright in the bedroom at night.” [ID 71] “Couldn’t sleep with it in the room.” [ID 15]

### EMA App

A total of 20 (50%) participants indicated that the EMA was easy to set up and use, fast, functional, and reliable in that it “worked the way it’s intended” (Table 2). Participants identified several challenges to using the EMA including technical issues and survey timing. Technical issues included app malfunctions (n=2, 5%), notifications without any clear air quality triggers (n=5, 13%), duplicate notifications (n=15, 38%), frequent need to sync the app (n=4, 10%), and surveys that continued asking about trigger events when the participant had indicated they were not home (n=5, 13%). The timing of EMA survey notifications was another identified challenge. In total, 8 (20%) participants stated that they received surveys at inconvenient times including during the night. Two (5%) participants noted they received surveys in the morning while they were still sleeping. Seven (35%) participants indicated they were unable to determine the source of the environmental exposure that triggered the EMA survey. Other EMA challenges included confusion regarding the survey questions’ skip logic (n=2, 5%) and the lack of open-ended options to provide a more thorough response to a survey trigger (n=2, 5%).

**Table 2:** Themes and quotes related to participant experiences with the ecological momentary assessment app.

EMA^a^ themes	EMA quotes
Easy to use	“The apps are really self-explanatory, which is really nice.” [ID 86] “It’s fast and easy; quick to load and simple to use.” [ID 9]
Notification timing	“I have irregular hours and sometimes I was asleep when the EMA sent a message, and sometimes I would miss it then.” [ID 4] “I received air quality alerts after midnight and before usual waking hours.” [ID 89]
Survey ambiguity and confusion	“The surveys were confusing to answer straightforwardly, and I wasn’t always sure I was being thorough or answering in the right way.” [ID 58]
Technical issues	“For part of the time, the app was not working.” [ID 4] “Getting multiple prompts for surveys with the same questions.” [ID 120] “I almost always had to ‘sync’ before it would show a survey on the page.” [ID 11]

^a^EMA: ecological momentary assessment.

### Home Spirometer

Overall, participants indicated that setting up the home spirometer was easy (n=17, 43%) and the device was easy to use (n=13, 33%; Table 3). Two (5%) participants noted that they liked the size and portability of the spirometer. Eight (20%) liked getting the real-time results of their lung function. Participants also identified challenges to using the spirometer. Thirty (75%) participants found the spirometer challenging to use for many reasons including the spirometer required too many breathing efforts, the instructions were tedious, and the spirometer did not register an effort correctly. Seven (18%) participants wanted more information on how to interpret the breathing test results. Well-documented side effects reported from 9 (23%) participants about spirometry assessments included lightheadedness and shortness of breath. Other comments included issues with a low battery (n=2, 5%), general dislike of using the device (n=2, 5%), and lack of clarity in the ZEPHYRx app instructions regarding when they were to use the spirometer (n=4, 10%).

**Table 3:** Themes and quotes related to participant experiences with the spirometer.

Spirometer themes	Spirometer quotes
Easy to use	“Very lightweight, easy to carry and clean.” [ID 58] “Spirometer and app were easy to use.” [ID 71] “I really liked that it worked well. It was compatible with my phone...that was easy to use in that way.” [ID12]
Real-time results	“I like that there’s an app that pairs with it and that you can get like feedback about, you know, your asthma pretty, pretty quickly.” [ID 71] “Really useful for me and I think I like it will definitely be more a bigger part of my sort of daily routine going forward.” [ID 58] “I noticed the results, after I would finish using it each morning. So I definitely paid attention to that. And I’m, and I think because of using it, am more aware of what my breathing looks like.” [ID 12]
Challenging to use	“Instructions for breathing tests are difficult.” [ID 41] “Almost always took me 6-8 tests to get the result, which felt like a lot.” [ID 93] “Once in a while it won’t catch my breath. Like it won’t like register that I’ve done it and it’ll need to do it again.” [ID 55] “I have had a hard time exhaling as much as they want me to.” [ID 60]
Clarity of results	“Didn’t necessarily know how to read the numbers.” [ID 45] “If there could been some type of brief explanation about numbers that were seen, that would’ve been great.” [ID 101]
Adherence	“It’s pretty easy if I could just remember to do the spirometer.” [ID 95] “Not always convenient to use the spirometer.” [ID 4]
Side effects	“Doing spirometry so often irritated my asthma.” [ID 71] “Exhausting to do multiple trials.” [ID 96]

### Overall Study

A total of 11 (28%) of the 40 participants indicated that the study was easy. Others noted that the study was interesting (n=18, 45%), educational (n=8, 20%), and useful for planned or already implemented behavior changes (n=5, 13%). Five (13%) participants reported an increased awareness of their indoor air quality and its relationship to their asthma symptoms. Those same 5 participants stated they had either made plans to implement or had already implemented behavior changes such as discontinuing or limiting the use of candles (n=1, 3%), using smaller amounts of certain products (n=2, 5%), and taking new actions when noticing their indoor air quality was poor (eg, opening a window to increase ventilation; n=1, 3%). Four (10%) participants did not notice an impact on their asthma symptoms or severity. Others said the study helped them take their medication daily (n=1, 3%) or hold their breath longer (n=1, 3%), and that it had generally improved their asthma symptoms (n=4, 10%). In total 10 of the 40 (25%) participants also stated that the components of the study worked seamlessly together and 16 (40%) indicated that the research staff were nice and helpful. Several participants noted that overall, the study worked well (n=7, 18%), was “cool” or “fun” (n=4, 10%), and was well thought out (n=3, 8%; Table 4).

**Table 4:** Themes and quotes related to participant experiences with the overall study.

Overall study themes	Overall study sample quotes
Increased awareness	“It actually made me more aware of what the triggers were that I never even thought about then I realized which cleaners I’m a little bit more sensitive towards.” [ID 13]
Behavior changes	“It was helpful to be able to see the values to know how they are affecting the quality of asthma and how to improve values that were able to be manipulated with environmental changes.” [ID 86]
Educational	“I do think it definitely gave me more information. Helped me understand what all could affect the air quality in my home. So it certainly was informative.” [ID 12]
Study implementation	“It felt like it was pretty seamless and, and easy to use. I thought it was a nice use of technology.” [ID 12]
Asthma impacts	“I don’t think it made a difference in my asthma.” [ID 37]
Anticipation of study results	“At the end of the project [I] would like some feedback and info about what we discovered and the results of the study.” [ID 4]
Recommendations	“There was a lot of paper in there, kinda overwhelming at first. I think it would’ve been less overwhelming if there would’ve been...just some bullet points showing what all the paperwork was.” [ID 7]

### SUS Scores

A total of 37 (92.5%) participants completed the modified SUS after the 14-day data collection period. The average scores for all of the subscales, as well as the item addressing overall integration of study devices, were above a 68 cut point, denoted by Lewis and Sauro [40], and thus are considered above average usability. A curved grading scale described by Lewis and Sauro [40] indicates that all of the subscales were graded at B or above with an A– grade for the overall integration of the 3 assessed devices (Table 5).

**Table 5:** SUS^a^ scores and grades per subscale.

Modified SUS subscale	Score, mean (SD)	Grade^b^
Home spirometer	75.9 (20.5)	B
Air Quality Monitor	83.9 (14.9)	B+
Ecological momentary assessment	74.8 (17.8)	B
Overall integration of devices	87.2 (20.9)	A–

^a^SUS: System Usability Scale.

^b^84.1-100=A+; 80.8-84.0=A; 78.9-80.7=A–; 77.2-78.8=B+; and 74.1-77.1=B [39].

## Discussion

### Principal Findings

This study aimed to evaluate the acceptability and usability of 3 devices used simultaneously in a feasibility study exploring real-time residential TVOC and PM_2.5_ exposures and real-time asthma symptoms, as well as lung function in adults with uncontrolled asthma. The initial development of the study included an intensive, iterative testing period while integrating the technologies used for the study. The research team spent more than a year testing the equipment and apps in trials to ensure participants would have a positive experience. Each modification made by the research team, in addition to mandatory app software updates, required an additional testing period to verify that all devices were working properly, step-by-step instructions were clearly and accurately written, and participant burden was minimized.

The integrated devices consisted of the Awair Omni indoor air quality monitor, the ZEPHYRx home spirometer, and PiLR EMA app. A 3-day, run-in period was an integral component of the study that allowed the participant to acclimate to the study devices and EMA surveys and allowed the research team to monitor for any problems prior to the 14-day data collection period. Two types of problems were identified—Wi-Fi connectivity and participants inadvertently logging out of the EMA and spirometer app. We were able to address the Wi-Fi connectivity issues on an individual basis. We added information to our orientation sessions informing participants not to log out of the EMA and spirometry app.

Participants were asked to keep their air quality monitor in the room they occupied the most in their home and to move it to their sleeping room at night. All participants received a minimum of 3 EMA surveys per day, including 1 morning and 2 random surveys. The morning survey addressed nighttime asthma symptoms (if present), and the random surveys queried about residential exposures, as well as asthma symptoms during the day. Additional EMA surveys occurred when elevated residential TVOCs or PM_2.5_ triggered an EMA survey in which participants were asked to use their spirometer.

The acceptability of both the devices and the study procedures was positively conveyed by participants based on the 7 and 14-day interviews and responses to the open-ended SUS items. The indoor air quality monitor was considered acceptable given the positive comments regarding the ease of use, educational capabilities, and reporting of air quality findings. While some technical issues were noted by participants, these were addressed by the research team when identified. Our participants indicated that the EMA was easy to use, fast, and reliable, which is similar to findings from Nichols et al [29]. Common weaknesses of the EMA were also identified by our participants including technical issues, as well as concerns with survey timing or too many surveys [24,30-34]. In addition, our participants noted that the EMA survey would continue to query about residential exposures even when they responded that they were not at home. While the team tested this aspect of the EMA survey extensively, we found additional coding issues were due to updates in the operating systems of smartphones or application software.

Participants voiced mixed responses to the acceptability of the home spirometer. Similar to other studies, many participants reported that the setup of the spirometer was easy, the device was easy to use, and having real-time lung function results was positively viewed [21-23]. However, some participants voiced several challenges to using the spirometer including tediousness, difficulty with the breathing tests, and physiologic side effects, such as lightheadedness and shortness of breath. During the study, research staff noted that some participants were making 6 or more assessment attempts (consisting of deep breaths) to obtain a reading that was “acceptable” according to the spirometer. We then revised our orientation instructions to indicate that no more than 3 deep breaths should be attempted, irrespective of whether a reading was deemed acceptable. Given the issues with the spirometer, we recommend that a peak flow meter be considered as an objective measure of lung function in future work.

When asked about the overall study acceptability, participants provided positive feedback. Several noted that it was educational and had provided the impetus for them to initiate behavioral changes such as limiting the use of candles and potentially toxic products. While several participants stated that they felt participation in the study improved their asthma symptoms, others noted no impact on their asthma. Other studies [12] have reported less than 10% of those with asthma experienced immediate symptoms after an air quality event exposure. Similar to the app developed by Kim et al [41] for children, future research should expand the focus to include an app for adults with asthma to know, in real time, the indoor and outdoor air quality that may be impacting their asthma symptoms and promote mitigation behaviors and address their asthma symptoms. Future studies may also include an app that in real time examines exhaled breath as a biomarker of VOC exposure [42,43].

### Limitations

Our study had several limitations. Our sample was relatively small (N=40) and participants were primarily women, well-educated, and homeowners. As previously noted, smartphone and application operating system updates were a challenge. Further, the in-app spirometer onboarding instructions were modified by the company without notification. When these updates occurred, study staff had to quickly identify any discrepancies in the orientation materials and modify instruction sheets as needed. An additional study limitation was the inclusion requirement that participants have Wi-Fi access in their homes. Future studies should provide a mobile hotspot when Wi-Fi is not available. Finally, given the challenges with the spirometer, the validity of these data is of concern for those individuals who were unable to provide repeatable measures.

### Conclusions

This pilot study used a sample from 18 states to assess the acceptability and usability of the Awair Omni indoor air quality monitor, the ZEPHYRx home spirometer, and PiLR EMA app in determining real-time residential air quality environmental exposures, concomitant with real-time asthma symptoms and lung function, in adults with uncontrolled asthma. Participants found all study devices easy to set up and use. Concerns were raised related to the side effects of spirometry assessment and the receptiveness or technical issues of the EMA surveys. Based on the modified SUS, usability was determined to be acceptable for all study devices, as well as the overall study. Recommendations for future studies include increasing the sample size and diversity of participants and expanding troubleshooting efforts to ensure minimal disruptions to the data collection process.

## Abbreviations

CAB: community advisory board
EMA: ecological momentary assessment
PM2.5: particulates 2.5 microns or less in diameter
REDCap: Research Electronic Data Capture
SUS: System Usability Scale
TVOC: total volatile organic compound
VOC: volatile organic compound

## Data Availability

The data sets and analyzed during this study are available from the corresponding author on reasonable request and contingent on Institutional Review Board approval.
